# What are the common areas of risk and their characteristics found in intermediate care from an occupational therapy perspective? A scoping review

**DOI:** 10.1177/03080226221079233

**Published:** 2022-04-29

**Authors:** Craig Newman, Phillip Whitehead, Mary Thomson

**Affiliations:** 1 Social Work, Education and Community Wellbeing, 5995 Northumbria University, Newcastle upon Tyne, UK; 2 Population Health Sciences Institute Faculty of Medical Sciences, Newcastle University, UK; 3 Newcastle Business School, Northumbria University, UK

**Keywords:** Occupational therapy, intermediate care, risk, safety

## Abstract

**Introduction:**

Engaging with risk is a certain and unavoidable part of occupational therapy. Intermediate care services are mostly accessed by older people with complex needs, yet little is known in the literature about the extent, type and nature of risk involved in these services.

**Method:**

A scoping review was systematically conducted to map the common areas of risk (risk domains) from an occupational therapy perspective. Thematic analysis was conducted in order to identify the risk characteristics related to the literature reviewed.

**Results:**

25 journal articles were identified and arranged into 10 risk domains: Falls, discharge, practice errors, activities of daily living, pressure care, frailty management, patient handling, loneliness, nutritional care and language barriers. Three risk characteristics were identified: (1) Risk awareness and identifying risk, (2) decision-making under risk and (3) improving safety.

**Conclusion:**

Occupational therapists play a diverse role in migrating risk for older people which is not fully explored beyond addressing deficits in functional ability and hazardous environments. The process of how risk is controlled and reconciled with occupation and how positive risk-taking is facilitated are implicit and not directly addressed within the literature reviewed. The findings reveal gaps in knowledge and provide a foundation for further research.

## Introduction

Intermediate care is a short term intervention that occurs between primary and secondary care and is mainly accessed by older adults with complex needs. It is internationally recognised as a healthcare model and is predominantly focused on maintaining a person’s independence in their home by avoiding unnecessary hospital admissions and premature residential care (NHS Benchmarking Network, 2017). Intermediate care can include inpatient facilities which offer rehabilitation and convalescence as a step to transitioning to home or other care arrangements. As part of its provision, it prevents and reduces risks, errors and harm as part of patient safety. Patient safety is a healthcare discipline that is concerned with services provided during the provision of healthcare (NHS, 2021). Post such provision requires the management of risk through an occupational therapy risk enablement plan so that a person can carry out and benefit from their activities safely (RCOT, 2017).

Risks are normally associated with harm and whether considered or unconsidered they are everywhere; at home, at work and in both activity and inactivity (Carson and Brain, 2008). Morgan (2004 p. 18) defines risk as ‘the likelihood of an event happening with potentially beneficial or harmful outcomes for self and others’, thus emphasising both positive and negative aspects of risk-taking. In occupational therapy, negotiating the safest approach to risk-taking is an intrinsic part of a service user’s progress (RCOT, 2017).

Determining the nature of a risk and the opportunity it may or may not present is a cognitive process which includes subjective viewpoints (Gallagher, 2013; Breakwell, 2007). These cognitive processes also include some less obvious psychological factors which are related to how we make judgements in conditions of uncertainty, namely, the effect of heuristics and biases (Breakwell, 2007; Trimpop, 1994). Clinical and professional reasoning involves making judgements on risk-prone situations and occupational therapists use informal theories and tacit knowledge in their decision-making (Carrier et al., 2010). Heuristics can provide a mental shortcut to problem-solving, thereby reducing cognitive burden, but it can also lead to unhelpful bias like risk avoidance which can encroach on the ethical principles of autonomy, beneficence, non-maleficence and justice (Carson and Brain, 2008; Schell and Schell, 2008). There is a duty of care to support clients to take measured risks in occupational therapy (RCOT, 2017), this is sometimes clouded by fears of accountability and blame (Morgan, 2004). Carson and Brain (2008) contends that failing to support risk-taking can lead to serious consequences for those receiving care and avoiding risk-taking where there is a duty of care is not a guaranteed way to avoiding a harmful outcome or liability.

Effective risk management is achieved as a result of, and attention to, its preceding factors, which commonly include awareness, identification, assessment, action, communication and review to ensure harmful risk is minimised and positive therapeutic benefits are enhanced (Gallagher, 2013; Haxby et al., 2011; RCOT, 2017). The risk management process becomes particularly challenging when those with complex needs transition between higher dependency care to lower dependency care arrangements or where higher dependency care can be avoided in favour of more suitable support (National Institute for Health and Care Excellence, 2017). Additionally, the therapeutic use of risk is subject to client agreement and those that have mental capacity can choose their level of compliance or refuse such interventions which mitigate risk. Engaging in activity that presents a significant risk of harm where risk cannot be reduced to a reasonable level also presents complexity for occupational therapists. As such, refusing to support such an activity can be appropriate providing a person is made aware of all the risks and the activity is made as safe as possible. Making decisions like these is also subject to determining a client’s mental capacity and where there is a belief that capacity is lacking, risk-taking should be approached on a decision per decision basis and proportional to the level of understanding of the service user (RCOT, 2017). Such challenges are commonplace in intermediate care delivery.

In the United Kingdom, intermediate care and reablement provision are divided between home-based, reablement, bed-based and crisis-response services (National Institute for Health and Care Excellence, 2017). These services are accessed mostly by older people aged between 79 and 90 years (NHS Benchmarking Network, 2017). Demand for intermediate care is increasing as the 85+ age group is the UK’s fastest growing population and is set to double to 3.2 million by mid-2041 and treble by 2066 (NHS Benchmarking Network, 2017; ONS, 2018). As age increases, so does the likelihood of incurable long-term illness such as diabetes, cardiovascular and chronic respiratory disease (Wright et al., 2017) and ill health arising from multi-morbidity, frailty, dementia, malnutrition, falls and hip fractures, mental health problems, sensory loss, loneliness and social isolation (Age UK, 2019). These considerations together with a multitude of extrinsic factors (e.g. resource availability) require complex decision-making under risk to enable safe risk-taking. In intermediate care, positive risk-taking has become a prominent risk contingency principle. Positive risk-taking is ‘…balancing the positive benefits gained from taking risks against the negative effects of attempting to avoid risk altogether’ (National Institute for Health and Care Excellence, 2017, *p*. 17).

Health and wellbeing involves more than just the absence of disease. Occupation is a contributing factor to wellness and is fundamental to how people realise aspirations, satisfy needs and cope with the environment (Wilcock, 1993). Mcintyre and Atwal (2005) contend that occupational therapists evaluate and assess multiple pathologies in old age together with social, psychological, spiritual and cultural factors, which present profession-specific challenges. Risks in intermediate care are complex and heterogeneous and the literature from an occupational therapy standpoint is limited. The purpose of this scoping review is to identify studies conducted in intermediate care settings which relate to occupational therapy risk management in order to pool the available research and map key concepts. This scoping review aims to

1. identify the common areas of risk in intermediate care from an occupational therapy perspective;
2. provide insight into these common areas of risk (risk domains) by establishing their volume and scope from the available research and
3. identify the nature and characteristics of the risks in the research reviewed.

## Method

A scoping review was conducted in order to meet the study aims and to map the key concepts in this area, including the main types and sources of evidence available (Arksey and O’malley, 2005). The framework, in accordance with the recommendations by Arksey and O’malley (2005), was implemented using the guidance of the Joanna Briggs Institute of Applied Health Sciences and McMaster University, manual for scoping reviews. This framework and guidance assisted in identifying the research question, identifying relevant literature, study selection, charting the data, and collating, summarising and reporting results.

### Identifying the research question

The research question was developed by preliminary database searching and the initial reading of relevant literature. The research question was constructed using a collaborative process between the authors and identified as: What are the common areas of risk and their characteristics found in intermediate care from an occupational therapy perspective?

### Identifying the relevant literature

A systematic search using the databases CINAHL, PubMed, AMED and MEDLINE was conducted in December 2019. A three-stage search strategy was implemented, and regular team meetings were held between the authors to develop a search protocol. This included an initial search using keywords in the titles and abstracts in the retrieved records, a second stage to search the databases using the same identified keywords and a third stage to screen the reference lists of the included studies. Searches were not restricted by date, publication type or by non-peer review and non-English language studies were included. A search string was created using the divisions of occupational therapy, intermediate care services and risk and the variations of criteria therein (see Table 1). Boolean operators, truncation, wild card and proximity features were adjusted when necessary for each search and MeSH indexing was either not available or limited and was not used.

**Table 1.** Search terms.

No. of terms used	Search techniques
Occupational therapy (n=1)	Occupational therap*
—	AND
Risk (n=11)	Risk* OR threat* OR harm* OR hazard* OR danger* OR endanger* OR safe* OR accident* OR expos*OR uncertain* OR vulnerab*
—	AND
Intermediate care (n= 23)	Intermediate care OR reablement OR re-ablement OR home* OR bed* OR rehab* OR comm* OR restor* OR integrat* OR crisis* OR rapid* OR satellite W2 team OR inreach OR in-reach OR safe W2 haven OR mobile W2 rehabilitation OR recuperat* OR transitional W2 care OR three W2 tier OR emergency W3 team OR emergency W3 teams OR evercare OR discharge*

W2 and W3 = word proximity to adjacent word.

### Study selection

**Figure 1.**
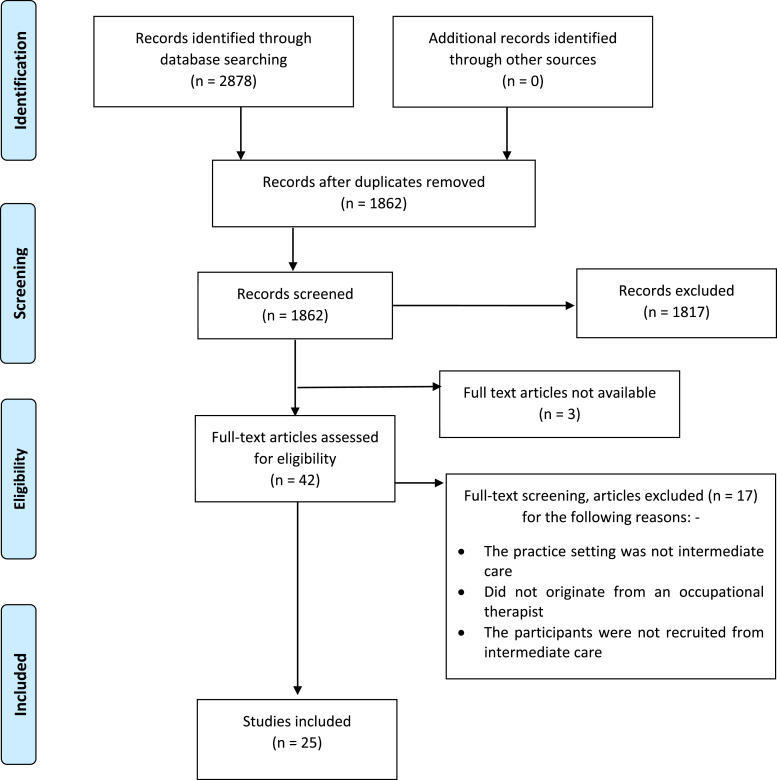
The article selection process using the PRISMA flow chart (Moher et al., 2009).

All records identified from the databases were uploaded to EndNote X9 and duplicates were removed. To be included, articles must have originated from at least one post-registered occupational therapists’ perspective, be within the remit and/or definition of intermediate care and include an aspect of risk management. These perspectives included clinical and professional reasoning/decision-making, opinion, perceptions and reflections. The National Institute for Health and Care Excellence (2017) core guidelines, the National Audit of Intermediate Care (2019) and Grant et al. (2007) provided intermediate care definitions. Risk terminology was identified in the Royal College of Occupational Therapists, ‘Embracing risk, Enabling choice’; Department of Health’s, ‘Best Practice in Managing Risk’ and ‘Independence, choice and risk: a guide to best practice in supported decision making’ guidance (DOH, 2007; DOH, 2009; RCOT, 2017). A decision tree (Appendix 1) was developed for the purposes of applying criterion. Duplicate EndNote files were created for two authors to screen the titles and abstracts independently. The results from each of the reviewers’ screening were combined into one EndNote file and the first author completed a full text review of each study. Meetings between the reviewing authors were held to resolve screening discrepancies; three areas of exclusion were applied, as shown in Figure 1. The studies that were subject to screening discrepancies and/or required further review for inclusion or exclusion were screened by full text by the authors independently before agreeing on exclusion. Studies that did not meet these criteria or were associated solely with primary acute care discharge were excluded; however, studies that did not specify the exact discharge setting and/or included both acute care and rehabilitation occupational therapy perspectives were included. Studies were included where occupational therapists were part of multi-professional groups of participants. Studies from the perspective of occupational therapy assistants or students were only included where all the other inclusion criteria had been met. Additionally, studies were not excluded based on whether a person had a particular condition, such as stroke or dementia and/or their particular circumstances that is, prison, temporary or residential accommodation, as per Sec. 1.3.2 of the National Institute for Health and Care Excellence (2017) intermediate care core principles.

### Assessment of methodological quality

An assessment of the methodological quality of included studies was undertaken, in accordance with the recommendation from Unsworth (2020) for occupational therapy scoping reviews, as shown on Appendix 2. For the qualitative and quantitative studies, this was conducted using the McMaster University critical review tools (Law et al., 1998; Letts et al., 2007). For the other study designs, the mixed methods and Delphi study were assessed using this critical review criteria for their qualitative and quantitative methods and critical appraisal guidance from Aveyard (2019) was used in relation to critiquing the literature reviews included in this study. This was completed for all included studies by the first author. Eight studies (32%) were selected randomly and screened independently by the second author to confirm the accuracy of their appraisal. Appraisal discrepancies were discussed during a team meeting between authors, whilst there was a high level of agreement in most areas the first author rechecked areas relating to the reporting of statistical significance in all quantitative studies and the reporting of the decision trail and four components of trustworthiness in all qualitative studies. Surveys which yielded quantitative and qualitative data were assessed using the quantitative tool. Assessment of methodological quality of the qualitative and quantitative studies is summarised in text and a table, the other study designs are summarised in text only.

**Table 2.** Study summary.

Risk domain	Author(s), year	Methodology/publication description	Study purpose	Location/sample	Key findings	Limitations (reported)
Activities of daily living	Gooch, 2003	Quantitative (postal survey) British Journal of Occupational Therapy	To describe the bathing assessment methods used by occupational therapists when working with adults with physical disabilities and to explore the factors considered important during the assessment and solution phases of bathing intervention.	UK (NHS and Social Services in Greater London) 90 of 108 respondents Occupational therapy (n=90) 55 completed by NHS staff 35 completed by social services staff 83.3% response rate.	Methods of assessment used: •Client observation at home without water (n=85) and face-to-face client interviews were the most used assessments (n=83). •Over 50% of the respondents indicated they used their own assessments. •Telephone interviews (n=20) were selected more than standardised assessments (n=15). Factors considered during the assessment: •Mobility (n=89) and safety (n=89) were selected by nearly every respondent. •NHS respondents selected client priorities as a factor, not ranked so by Social Service (SS) respondents. •Safety factored higher than medical diagnosis for NHS respondents, whilst the SS respondents considered the latter more important. Factors considered during the solution stage: •Client disability was selected by all respondents (n=90) followed by client priorities (n=87) and environmental factors (n=83). •Clients’ priorities was again selected by NHS respondents higher than SS respondents who selected client disability slightly higher than the NHS staff. •The NHS group attributed some importance to equipment availability. The SS group attributed some importance medical diagnosis.	•Generalising the results – sample was limited to Great London. •Independence of the practitioners cannot be assumed (linked to one organisation). •Reliability of the questionnaire (solely produced for the study).
Activities of daily living	Carrier et al., 2010	A scoping review Australian Occupational Therapy Journal	To synthesise current knowledge about community occupational therapists’ clinical reasoning (CR) in determining interventions important to the ability to live at home.	Australia	The final analysis was performed on 15 textbooks and 25 articles (n = 19 on occupational therapists’ CR, n =6 on community occupational therapists’ CR) The community occupational therapists’ studies (n=6) revealed five key elements: •Cognitive processes (problem-solving) underlying CR (n =3; 50%). Two different strategies identified hypothetico-deduction and pattern recognition. •Dimensions of CR (n = 4; 67%). Identified as scientific, diagnostic, procedural, narrative, pragmatic, ethical, interactive and conditional. Frequently used simultaneously. •Factors influencing CR (n = 6; 100%). Factors identified as being internal and external. Internal are the therapist’s expertise and personal context. External factors were the client and practice context. •Methods used to document CR (n = 6; 100%). CR knowledge development is influenced by the methods used to study it, predominantly protocol analysis (case studies, observations) and interpretative methods (grounded theory). •Elements of community occupational therapists’ CR still unknown (n = 4; 67%). How community occupational therapists integrate tacit and formal knowledge is still largely unknown.	•A scoping review does not provide an assessment of the quality of the studies examined. •Information not identified, as textbooks are not systematically included in electronic databases. •Searches could have covered a longer period with more CR based terminology.
Discharge	Moats and Doble, 2006	Literature review Canadian Journal of Occupational Therapy	To review the literature regarding the decision-making process of discharge and how autonomy and risk avoidance factors influence these decisions for occupational therapists.	Canada	Factors that influence risk avoidance: •Social values, service traditions, legal pressures and political and economic directives. Ageism also supports risk avoidance. •Conflicting ethical principles of beneficence and autonomy may result in persuasive methods to resolve ethical dilemmas. •Family members may fail to respect risk-taking choices of the elderly in fear of health workers condemnation/legal reprisals. Risk elimination may be preferred but does not justify therapeutic paternalism. Autonomy and risk-taking: •Anti-paternalistic decision-making preferred. •Autonomy is dependent on context (legal, medical). •Risk avoidance can contribute to loss in self-worth, identity integrity and control. Older person homes take on a large significance and provide a sense of identity. •The traditional medical ethics perspective can fail to understand the concept of autonomy in full. Autonomy considerations in decision-making of informed consent, whilst appropriate for acute care, maybe insufficient in respect of long-term decision-making Occupational Therapy and client centre practice •Guided by autonomy promotion and accepting the risk a client is prepared to take. •Client-centred practice ideals are often abandoned when clients place themselves in danger. Collaboratively balancing risk avoidance and autonomy is required. •Clients should participate in decision-making congruent with their abilities/cognitive level. •Careful and considered negotiation of risk avoidance can maximise autonomy abilities in the future and together with extended rehabilitation, community-based support and continued care can facilitate better outcomes. •Barriers to adopting a negotiated approach are often systemic in nature and outside of the immediate control of an individual practitioner.	•None reported
Discharge	Moats, 2007	Qualitative (semi-structured interview) Canadian Journal of Occupational Therapy	The study explored occupational therapist discharge decision-making models and their relationship with the professional issues of client-centred practice and enabling occupation for older persons.	Canada (acute and geriatric and specialised rehabilitation) Occupational therapists (n=10)	Three themes were identified: Being client centred •Therapists support client centre practice and included family as the ‘client’ from two perspectives: 1) Involvement as caregivers and 2) needed as proxy decision makers. •Client centeredness became difficult when family unwilling to accept risk. •When the client was not competent or where family involvement was minimal, therapists recognised a need for increased professional involvement. •Cognitively impaired but not officially ‘incompetent’ were recognised as complex and ill defined. Therapists struggled with risk situations where these decisions had to rest with the client to be client-centred practice. Style of decision-making. •Client-centred practice can involve blending client defined, professionally driven and negotiated styles of decision-making. Sometimes in this discourse, there was evidence of the use of intimidation, persuasion and coercion. •One way that some therapists defined their practice as being client-centred was by insisting they only make recommendations, not decisions. Occupations and the importance of home. •There is value to doing occupations in a familiar environment and there is a power dynamic shift in favour of the client. •With practice time constraints, home visits can be overlooked. •Therapists focus on occupations an older person is no longer safe to do and not future occupations during decision-making. Additionally, a negotiated model of decision-waking proposed to enable decision-making processes.	•The full saturation of data was not achieved as findings were based on single group of interviews with a small number of therapists, because of this the proposed model will need testing and further development. •This researcher’s biases may have influenced interpretation of the data.
Discharge	Nygård et al., 2004	Qualitative (focus group and interviews) Scandinavian journal of caring sciences	To investigate the perceptions of therapists and clients on common practice home assessments and interventions prior and post discharge from a geriatric inpatient clinic.	Sweden (geriatric inpatient care) Occupational therapist (n=9) Participants (n=23)	Client problems and occupational therapy interventions documented on the pre-discharge home visits. Problem (n=107) frequencies: •Most frequent was motor capacity which obstructs activity or involves safety risk (82/107) •Inadequate cognitive/psychological capacity obstructs activity or involves safety risk (6/107) •Explicit obstacles in physical environment (17/107) •Incapability to perform certain activities (2/107) Frequencies of therapist interventions (n=136) •Assistive devices/housing adaptation (76/136) •Contact with secondary person (25/136) •Information/recommendation (20/136) •Removing environmental obstacles, rearranging furniture (10/136) •Instructions for adapted methods of for example transfer (5/136) Clients’ evaluations of interventions (n= 130) •Situations where the client was explicitly satisfied (73/130). •Situations where the client had an alternative (45/130). •Situations where the client was dissatisfied (11/130). •Situations where the client was partly satisfied (5/130). Occupational therapists were generally in agreement with the client’s responses except when putting themselves at risk. Pre-discharge home visits were important for the clients’ safety. Temporal delay (delivery, installation of equipment) caused safety risks. Client renounced support because of the overabundance of health and social care persons on their home. Follow-up visits – time at home imperative for needs to be discovered and interventions to be adjusted.	•Data gathered within the priorities of clinical practice/client needs meant not all client problems were addressed. •Individual therapist interpretation in categorising the data may have affected the results. •Risk of bias, as therapists may have chosen to follow up their own clients. •The distribution of client diagnosis at the time of the study may have affected the outcomes.
Discharge	Davis Aisling and McClure, 2019	Quantitative (survey) Irish Journal of Occupational Therapy	This study aims to investigate current clinical practice during home visits and the value that occupational therapists’ attribute to home visits within an Irish context.	Ireland (acute, rehabilitation and convalescence settings) Occupational therapist (n=122)	Results from the quantitative section •44% completed 2–5 home visits per month, 8% completed 5–9 visits, 1.7% completed 10–14 visits and 0.8% completed 15+ visits per month. •50%+ reported taking between 1 and to 1 ½ to complete a home visit. 12 respondents reported 2 hrs^+^ •3% took less than 30 mins to write reports, 41% of the participants reported they take between 1 hr and 1½ to complete reports. •93% per cent of participants reported bringing a mobile phone, measuring tape and gloves on home visits as standard, 56% took a cardiopulmonary resuscitation mask. 9 respondents stated they took a personal alarm. •70% of participants provide between 5 and 10 recommendations post visit Results from the qualitative section. •Benefits of a home visit during discharge planning. A high number of participants identified the opportunity to assess patients within their own, familiar environment. The ability to identify potential difficulties, reduce falls risk and improve safety was also mentioned by several participants. •Most participants cited lone working as a significant risk during a discharge home visit. The risk of unknown social factors included aggression from family members and anger regarding service provision faults, unruly pets, poor hygiene, and houses in disrepair (holes in floorboards) and vermin. •Patient safety issues included falls risk or medical emergency as potential risks during a home visit. •Patient criteria for a home visit included living alone, falls risk, prolonged stay in hospital, changes or decrease in functional or cognitive status. •The improvements to discharge planning home visits (DPHV) included standardised checklists, assessments and policies governing DPHV practice, additional time to complete visits, additional resources, better transport options, occupational therapy assistant support, secretarial/admin back up and collaboration between community services and MDT. •Successful DPHV included ascertaining whether a discharge is suitable, safe and sustainable; non-suitability was also considered a success. Success on visits was also defined as the identification of risk factors and patient/family’s awareness of these factors following education.	•Reported practice, not observed practice. Therapists may be describing practice they espouse to and not representative of routine practice. •Participants were from the Dublin area; the findings may suggest a bias towards urban areas and therefore may limit the generalisability of findings nationwide.
Discharge	Simning et al., 2019	Quantitative (longitudinal study) Journal of the American Medical Directors Association	The primary objective of the study was to examine whether rehabilitation providers can predict which patients discharged from a skilled nursing facility (SNF) would be successful in their transition to home, controlling for sociodemographic factors and physical, mental and social health characteristics.	US (Two SNF rehabilitation units) Medical providers, occupational therapists, physical therapists and social workers (exact representation unknown)	The longitudinal study was conducted from March 2016 to November 2017 with English speaking patients aged 65+ years. 112 older persons (mean age 78.1 years) were recruited into the study. Patients were interviewed at 2 weeks upon admission, every 2–4 weeks during their stay and at 1 week, 1 month and 3 months post-SNF discharge. The dependant variable and outcome measure were ‘failed transition to home’ and the independent variables were the healthcare professional’s responses (predictions) and the patient’s sociodemographic factors and physical, mental and social health characteristics. A 7-point Likert-type scale from ‘strongly disagree’ to ‘strongly agree’ was used and dichotomised into ‘neutral or negative prediction’ and ‘positive prediction’. The healthcare professionals were asked to predict who would successfully transition to home. •The predictions of the occupational and physiotherapists were associated with the discharge outcomes. •The predictions of the medical providers and social workers were not associated with the discharge outcomes. The study suggests occupational, and physiotherapists may have unique insights into determining which post-acute rehabilitation patients will struggle with SNF to home transition.	•Study was not designed to test the predicative capabilities of the participants. •Precision of data - hazard point estimates, consider with caution. •The main outcome measurement is unique to the study. •2 SNFs were used – generalisability limited. •Dementia patients and those unable to provide consent were excluded. •The patients’ functional impairment, not known. •Speech and language pathologists’ data limited and not used.
Frailty	Roland et al., 2011	Mixed methods Qualitative Repertory grid-guided interviews. Quantitative Participants were asked to rate their answers using a 7-point scale. Physical & Occupational Therapy in Geriatrics	The study’s purpose was to explore physical and occupational therapists’ perspectives of ‘frailty’ within their community practice, and to develop a definition of how they view and manage frailty in their practice.	Canada (home and community centre) Occupational therapist (n=4) Physical therapists (n=7)	There was a consensus among therapists to characterise frailty as deterioration in physical and psychosocial abilities making it difficult to complete activities of daily living (ADL), resulting in functional dependence and an inability to thrive. The primary areas of frailty were discussed (a) characteristics of frailty, (b) defining frailty and (c) managing frailty. Characteristics of frailty: •Physical – risk of falls, poor functional endurance and limited mobility. •Psychosocial – isolation, poor self-management and depression Defining frailty •Image of frailty – multiple components, complicated medical history, spectrum of severity. Managing frailty •Limited time to identify at risk populations and implement prevention strategies. •Responding to crisis situations whilst managing normal case load, unable to include follow-ups. •Therapists primarily focus was on observing clients at home and intervening to manage frailty. Most often home-exercise programs were implemented. •The involvement and collaboration with other healthcare practitioners. Other members of the client’s support network are also involved.	•Predetermined questions may have inhibited the insight into frailty. •Small sample and disproportionate representation of therapists.
Falls	Buri et al., 2000	Qualitative (Phase 1: semi-structured interview Phase 2: Observational study) British Journal of Occupational Therapy	To determine if perceptual dysfunctions in the elderly with cognitive impairment as an additional risk factor for falling and, if so, what types of perceptual dysfunctions pose the greatest risk.	UK (four residential homes) Phase 1: (purposive sampling) Occupational therapist (n=1) Physiotherapist (n=1) Phase 2: Researcher (n=1) Residents observed (n=unknown)	Phase 1: Three categories emerged as being important considerations to determining perceptual dysfunction contributing to the risk of falls in the elderly with cognitive impairment: •Interaction with the environment •Movement •Psychological factors Phase 2: Further subcategories were formulated and used to describe observed behaviour. •Interaction with the environment: colours and patterns, interior furnishings, negotiation of space, background noise and object recognition. •Movement: wandering, speed, pattern and accuracy •Psychological factors: fear/ lack of fear and spatial disorientation Spatial disorientation emerged as the most important perceptual risk factor.	•Small sample not generalisable. •Validity (trustworthiness) may have been affected by the subjective interpretations of the researcher. •Observation may have affected the residents’ behaviour.
Falls	Kinn and Galloway, 2000	Quantitative (postal survey) British Journal of Occupational Therapy	To investigate whether therapists do anything to prevent falls and, if so, whether they assess elderly people for their suitability to be educated in how to rise after a fall.	UK Respondents (n=145) Occupational Therapy (n=105) Physiotherapy (n=32) Home Care (n=3) Nursing (n=3) Social work (n=2)	Almost all (93%) of occupational therapists and physiotherapists confirmed falls was an issue they dealt with in the over 65 age group. The range of interventions used was categorised into three broad themes environmental, physical and education. Occupational therapists’ responses to the types of interventions used: •Environmental (64%) •Physical (25%) •Educational (10%) Physiotherapists responses to the types of interventions used: •Environmental (11%) •Physical (70%) •Educational (18%) Approximately half of the respondents (49%) assessed the ability of their patient to rise after a fall. Over half (54%) of the respondents had considered teaching or had taught people how to get up after a fall.	•The sampling method (convenience sample) produced unequal participant representation between the disciplines. This may attract criticism from a methodological perspective and interpretation of the results.
Falls	Ruchinskas et al., 2001	Quantitative (a two-part survey. Part 1: A self-reporting non-cued questionnaire. Part 2: A self-reporting cued questionnaire Rehabilitation Psychology	To examine the capacity of occupational, physical, Physiatry, recreation and speech therapy therapists to identify risk factors for falls.	US (three academic medical rehabilitation centres) 55 of 81 responded. Occupational therapy (n=14) Physiatry (n=12) Physical therapy (n=24) Recreation therapy (n=2) Speech therapy (n=3)	Both parts of the survey were compared to two empirically supported falls risk factors, advanced age, and history of falls. Part 1: •14% identified advanced age as a risk factor for falls. •5% identified history of falls as a risk factor for falls. Part 2: •11% identified advancing age as a risk factor for falls. •77% identified history of falls as a risk factor for falls. There were no significant demographical influences in how many times advanced age and history of falls was listed in either questionnaire. Additionally, there were no significant differences between disciplines on their ratings. The use of cueing helped therapists make a stronger prediction on the history of falls as a risk factor but not advanced age. Staff education on validated risk factors for falls may reduce the potential for errors and improve decision-making and patient care.	•Sampling bias, as a proportion of the respondents did not complete the survey in the allotted time. •Therapists may exhibit different behaviour and clinical judgements when treating patients in a rehabilitative setting.
Falls	Ruchinskas, 2003	Quantitative (prospective cohort study) American journal of physical medicine & rehabilitation	To assess the ability of physical and occupational therapists engaged in rehabilitation to predict falls in the elderly within a 3-month period after discharge.	US (rehabilitation unit) 15 months total duration Elderly patients (n=165) aged 60+ years identified during a 12 m period. Contacted (n=132) at 90 days post-discharge Physical therapists (n=14) Occupational therapists (n=7)	•Elderly respondents (n=16) 12% reported one or more injurious falls within the 3 m period post-discharge period. Considerably lower than the pre-discharge falls rate of 38% described by the respondents before admission. •Those who had fallen before admission had a higher likelihood of falling post discharge. •Statistical differences in the rate of falling between respondents with a recent neurological event (11 of 23) versus those patients with an orthopaedic or general medical diagnosis (5 of 109). •Occupational therapists predicted 13% and physical therapist predicted 20% as a high risk of falls, slightly greater than the 12% who reported falling. •Only seven of the fallers (44%) were rated as high risk by either of the disciplines. •Degree of strength, safety awareness and balance were most cited as salient factors in determining who was at high or low risk of future falls.	•Interpretation of the results – one cohort of therapists participated. •A disproportional number of patients with neurological disease were lost to follow-up (post discharge). •Increasing the follow-up stage at 3 months to 12 months may have improved predictive accuracy.
Falls	Woodland and Hobson, 2003	Literature review Canadian journal of occupational therapy	To review the current falls-prevention literature for community-dwelling older adults from an occupational therapy perspective, to highlight the important contribution occupational therapy could make to this functional problem.	Canada	•The literature identifies numerous risk factors involved for this population which can be categorised as intrinsic (personal) and/or extrinsic (environmental). •Occupational therapy appears to be underrepresented in the current falls-prevention literature, and therefore, the role of occupational therapy in this area may not be fully developed. •Using the Canadian Model of Occupational Performance to categorise the literature revealed some gaps in knowledge. Cultural, economic, political and legal elements of the environment tend to be overlooked. •Falls are also attributed to personal factors (cognitive, affective and physical) that can be modified. •Importantly, there is a clear gap in knowledge regarding the role occupational plays in precipitating falls. •Client-centred practice, compliance (client receptiveness and adherence to strategies) and follow-up (to monitor adherence and safety) were identified as important considerations to prevent falls among this population.	None reported
Falls	Olij et al., 2017	Delphi study (two rounds) Injury www.elsevier. com/locate /injury)	To determine a) how health professionals detect community-dwelling elderly with an increased risk of falling; b) which falls-prevention activities are used by health professionals and why; c) how elderly can be stimulated to participate in falls-prevention programs and d) how to finance falls prevention.	Netherlands Online Delphi study Round 1 68% (n = 85/125) Round 2 58% (n = 72/125) Participants included: community physiotherapists, community nurses, general practitioners, occupational therapists and geriatricians.	•Regular detection of fall risk of community-dwelling elderly with an increased risk of falling hardly takes place (median = 2 [hardly]; Inter Quarter Deviation (IQD) = 1). •The most important pitfall, was to reach community-dwelling elderly that are not in touch with health professionals (median = 5 [very important]; IQD = 1). •Involving informal caregivers was the most important success factor (median = 5 [very important]; IQD = 1) The panel was asked to indicate which health professionals should particularly be involved in detection of fall risk. •Consensus was reached concerning occupational therapist, being responsible for mapping fall risks in and around the house (n = 54/72; 75%). •According to 73% of the panel (n = 37/51), 0–40% of the elderly with an increased risk of falling are referred to exercise programs. Maintaining independence is the most important positive incentive to participate (n = 19/66, 29%). •Structural follow-up is often lacking. Physiotherapist was considered key in offering these exercise programs and follow-up. •According to the panel, health professionals that should particularly be involved in stimulating program participation are the general practitioner (n = 51/72; 71%) and the informal caregiver (n = 33/72; 46%). •Effective measures included medication monitoring, vision control and correction, and mapping fall risks in and around the house in falls-prevention programs. No consensus was reached on the effectiveness of screening for and supplementation of vitamin D. •The panel indicated a combination of national health education, healthcare counselling and removal of financial barriers would stimulate the participation of the elderly in falls-prevention programs.	•Guidelines on conducting a Delphi study are lacking. •The unequal distribution of professionals, as a large group of community physiotherapists and a small group of general practitioners participated, may have influence the results.
Falls	Hasegawa and Kamimura, 2018	Quantitative Hong Kong Journal of Occupational Therapy	This study aimed to develop a home safety assessment appropriate to be used by occupational therapists for the elderly with risks of falls in Japan, by adapting the Westmead Home Safety Assessment (WeHSA).	Japan 50 elderly people participated in the reliability study Occupational therapists (n=13) participated in this reliability study as therapist raters Occupational therapists (n=18) Participated in the validity study.	•49 items (69%) in the WeHSA-J were reliable and relevant for identifying fall hazards in the homes of elderly Japanese, these mainly involved activities of daily living with some simple instrumental activities of daily living. •The WeHSA-J generally had adequate inter-rater reliability, similar to the original WeHSA. Excellent or fair to good reliability was as follows, 65 items (92%) in the original version and 66 (93%) in the Japanese version. •Fifty elderly people (aged 78.2. +/- 7.1 years – 29 males (58%) and 21 females (42%) participated in this reliability study. •The most frequent hazards were identified as internal steps/stairs, seating, bathroom, bath and external steps/stairs. •The reasons for conducting a WeHSA-J on each participant were: a home visit before discharge from the hospital for 22 participants (44%), consultation on fall prevention for 17 (34%) and community-based occupational therapy services for 11 (22%)	•Small/convenience samples were used. •Majority of the occupational therapy raters in the validity study were hospital employees, therefore, the evaluation items accepted in this study might be appropriate for the impaired rather than all older persons.
Falls	Pighills et al., 2019	Mixed methods (medical chart audit, survey and focus groups) Australian Occupational Therapy Journal	The aim of this study is to identify factors that support the local adoption of best practice environmental assessment and modification (EAM) for falls prevention within a rural health service, from an occupational therapy perspective.	Australia (regional health service including Paediatrics, rehabilitation, home assessment and aged care via inpatient, outpatient or outreach services) Survey occupational therapists (n=14) Twelve of which participated in the focus groups (n=12) Patients’ charts (n=58) containing occupational therapy entries were used for the audit.	Twenty-four therapists were identified and 14 completed the survey (58.3% response rate). In accordance with the Integrated Promoting Action on Research Implementation in Health Services (I-PARHIS) framework. The results were categorised into 4 themes knowledge, attitude, confidence and experience. Knowledge: •Ten out of fourteen (71.43%) agreed there were no guidelines on best practice on environmental assessment for falls prevention. •All participants agreed (100%) people at a high risk of falls include those with a history of falls, visual impairment, those who are aged, have co-morbidities and have had a recent hospital visit. •Half of the survey respondents (50%) identified that they had attended additional formal courses on environmental assessment for falls prevention. Attitude: •78.57% strongly disagree and 21.43% disagree with preventing falls in the home is not a core concern for an occupational therapist. •64.29% agree and 28.57% strongly agree that they actively engage the patient and family in developing falls-prevention action plans. Confidence: •71.42% reported feeling confident in knowing when to conduct an environmental assessment for falls prevention. •85.71% agreed or strongly agreed that they felt confident in conducting a comprehensive environmental assessment for falls prevention. Experience: •100% responded that their current practice involved working with older people at high risks of falls. •64.29% had experience in completing pre-assessment screening to identify patient who may benefit from home assessment for falls prevention. •64.28% indicated that they carried out home assessments for falls prevention weekly. None of the charts audited documented a comprehensive process of hazard identification using a validated assessment tool or that an environmental assessment and modification for falls-prevention intervention was carried out. Focus group discussions identified three key themes which influenced uptake of EAM: confidence in, and awareness of evidence; key stakeholders’ support and knowledge of occupational therapy and perceived impact of time and resources required for implementation.	•A convenience sample was used to audit the medical charts. These were from regional occupational therapists who were more likely to provide EAM intervention for falls prevention. •In the audit, there was no documented evidence of the use of EAM to reduce falls risk; however, not including participant observation as part of the methodology may have resulted in a biased review of actual practice. •Focus group facilitator was not an occupational therapist. •Small survey sample.
Falls	Xu et al., 2019	Qualitative (focus groups) Disability and rehabilitation	The aim of this study was to investigate the perspectives of rehabilitation therapists on fall prevention programmes with community-dwelling stroke survivors in the Singapore context.	Singapore (rehabilitation) Occupational therapists (n=15) Physiotherapists (n=8)	Therapist perspectives were used to adapt the Stepping on After Stoke (SOAS) falls-prevention program. The qualitative data elicited from the four focus groups generated three main themes and sub-themes. Limitations of existing falls-prevention intervention for stroke clients. •Lack of a structured group-based falls-prevention programme for stroke clients •Lack of understanding of falls prevention after stroke among the caregivers. Adaptation of the Stepping On programme for stroke clients •Inclusion criteria for the SOAS programme. •Recommended changes •Additional key interventions/elements needed in the SOAS programme. Challenges in implementing fall prevention •Personal barriers •Social barriers •Organisational barriers •Cultural barriers Some common fall risk factors after stroke were suggested: medications (e.g. for hypertension), neurological visual disorder (e.g. hemianopia) and psychological disorders (e.g. post-stroke depression).	•Therapists (participants) had completed the generic Stepping On programme leader training and therefore are not fully representative of the therapists working with the stroke population in Singapore.
Language barriers	Squires et al., 2019	Qualitative (secondary data analysis) International journal of nursing studies	To explore home healthcare professionals’ perspectives about how workload changes from managing language barriers influence quality and safety in home healthcare.	US (large urban home healthcare setting) Occupational therapists (n=1) Physiotherapists (n=3) Nurses (n=31)	From the parent study, 142 discrete passages focused on workload, from the secondary data analysis the following themes were generated: •Conditions that contribute to higher workloads and longer working days. These ‘conditions’ included transitions (e.g. weekday vs. weekend admission, timely notification of limited English proficiency status), caseload, interpreter services usage, visit length, geography and continuity of care/language concordant visit. •Willingness to address language barriers. ‘Willingness’ reflected the overall sense of the providers’ concerns and triumphs expressed when putting forth the added effort to address communication barriers with limited English proficiency patients and families to ensure quality care. •Barriers contributing to workload when addressing language barriers in home healthcare. ‘Barriers’ consisted of policy, organisational, patient and provider level factors that contributed to increasing workload in home healthcare in ways that lengthened the workday and potentially detracted from care Subsequent choices showed proactive behaviours to manage increased workload shaped by their perceived risk of the threats posed by the quality of interpreter services. Integration of language access services across all points of service delivery will increase system costs; yet not adding language access services also increases costs because of the increased risk for errors related to communication problems.	•Data taken from one agency. •Qualitative study design means these findings cannot be generalised across similar practice settings.
Loneliness	Chana et al., 2016	Qualitative (semi-structured interviews) British Journal of Community Nursing	The aim of this study was to explore the attitudes of intermediate care team professionals regarding loneliness, to understand the perspectives of the broader organisation regarding loneliness and to understand whether there are specific barriers that may prevent actively detecting and managing loneliness.	UK (NHS community healthcare trust) Occupational therapists (n=3) Physiotherapists (n=4) Nurses (n=3)	Findings present as four key themes: the attitudes of intermediate care team professionals towards loneliness; the perceived attitude of the intermediate care team service towards loneliness; the perceived control of intermediate care team professionals in detecting and managing issues of loneliness; and suggestions for overcoming barriers. The attitudes of intermediate care team professionals towards loneliness. •A very relevant issue for intermediate care team clients. •Cyclical and complex relationship between physical health, mental health, and loneliness. • Identifying and referring loneliness are professional priorities but managing it is not. • Barriers to referring loneliness to other services. Perceived attitude of the intermediate care team service towards loneliness. •Loneliness is a low priority for the intermediate care team service. •Intermediate care team funded to meet commissioners’ requirement. Perceived behavioural control of intermediate care team professionals in detecting and managing issues of loneliness. •A conflict between personal and service attitudes towards loneliness in intermediate care team clients. •Patient barriers to managing loneliness. •Variability in health professionals’ ability to identify and address loneliness Overcoming barriers. •A need for training. •A need for objective assessment of loneliness. Some participants felt the referral process (independent and social care services) were overly bureaucratic, time consuming and unreliable. These services sometimes did not align with client needs. With large caseloads and time pressures, it was very likely that lonely clients were not being identified. Many felt loneliness was considered a low priority and influenced by care commissioners who set service performance markers by which the intermediate care team are assessed.	•Generalising the findings. The sample was representative of healthcare roles in the intermediate care team; it was small and was from a single healthcare trust. •The views represented in this study are likely to be from professionals with an interest in loneliness in their clients.
Nutritional care	Mole et al., 2019	Qualitative (semi-structured interviews) BMC Geriatrics	This study aimed to explore the experiences and perceptions of the nutritional care of people living with dementia at home from the perspectives of healthcare professionals and home care workers.	UK (healthcare professionals/home care workers residing in the South-West England) Occupational therapist (n=1) Social worker (n=1) Nurses (n=1) Dietician (n=1) General practitioner (n=1) Home care workers (n=2)	Seven interviews were conducted, as part of the interview a vignette was used. All participants (n=7) were shown and read the same vignette, which outlined a fictitious scenario of a husband caring for his wife with dementia at home. Four themes were generated: •Responsibility for care (7/7) •In it together (6/7). •Practice restrained by policy (5/7). •Improving nutritional care (5/7). The participants felt a responsibility for those living at home with dementia received adequate care and nutritional care was an important factor in their ‘duty of care’. The participants also recognised that the caregiver would need support. Challenges to providing nutritional care included limited time to spend with individuals, knowledge of appropriate food and drink choices and decisions to replace carer support with meal delivery to reduce cost. Suggested improvements included raising awareness of nutritional needs and developing training aids regarding nutritional care and dementia Providing adequate training regarding identifying nutritional risks, helping family carers make appropriate food and drink choices will help prevent the risk of malnutrition.	•Individual perspectives of the situation in the vignette may have resulted in participants giving answers that were expected of their profession. •Participants were recruited through the lead researcher’s professional networks, which may have affected the interview dynamics/results.
Patient handling	Darragh et al., 2013	Qualitative (focus groups) American Journal of Occupational Therapy	To determine how therapists have integrated and use safe patient handling (SPH) equipment in rehabilitation and how this use affects therapy practice.	US (inpatient rehabilitation) Occupational therapists (n=14) Physical therapists (n=14) Physical therapist assistants (n=4) Occupational Therapy assistants (n=1)	Three major themes were identified which related to the question of how the equipment is used in and affects rehabilitation: choice, potential and safety. Choice: •Equipment selection was based on the physical, behavioural and cognitive – perceptual characteristics of each patient; features of each device; time and environmental demands; and potential uses of each device. •Functional mobility was the most reported therapeutic use for SPH. Passive mobility was reported as the second most common use of SPH. •Some patients expressed fear of SPH equipment (lifts). •A minority of therapists expressed concern that lifts promote passivity or deemphasise transfer training. Potential: •Increased options in therapy, more was accomplished, and patients were able to be mobilised early in their recovery. •Equipment had benefits for bariatric patients, those with medically complex conditions, or who were dependent. •Therapists considered themselves a limiting factor in manual handling. With use of SPH equipment, therapists were no longer a limiting factor and patients could work to their potential. Safety: •Safety included the prevention of injury to therapists and/or patients and about patient falls, skin breakdown, or debilitation. •Overall therapists thought patients were safer with SPH equipment. Fall reduction was reported. •Equipment was used to facilitate less time in bed and as prevention for … ‘over-shearing the skin or preventing them from lying in bed not doing much, getting pneumonias’. •Patients experienced a greater sense of security with equipment. •Therapist’s experienced less fatigue, pain and strain. •Confined environments and patient equipment (e.g. drains, IV poles) contributed to difficulties with using the equipment.	•Generalization is limited because of the qualitative methodology. •Cultural and policy expectations of using SPH equipment in these practice settings may have influenced the participants.
Practice errors	Scheirton et al., 2003	Qualitative (focus groups) American Journal of Occupational Therapy	To examine occupational therapists’ responses to practice errors in physical rehabilitation settings.	US (four physical rehabilitation centres) Occupational therapists (n=35)	Five themes were generated: •(1) Concept of practice error: It is against our standards; (2) perceived causes of practice error: Not just an individual matter; (3) emotional responses: ‘I felt horrible’; (4) impact on practice: Doing things differently and (5) Management of practice error: Being honest and taking initiative. Occupational therapists valued the lessons learnt from their errors.	•Participants varied in age and experience. •Social desirability may have affected therapist perceptions/reflections during the focus groups.
Practice errors	Mu et al., 2011	Qualitative (focus groups) American Journal of Occupational Therapy	The aim of this study was to investigate the strategies to prevent or reduce practice errors used by occupational therapists who practice in physical rehabilitation and geriatrics.	US (physical rehabilitation or geriatrics) Occupational therapists (n=34)	Four overriding themes emerged from the data: •Strengthen orientation and mentoring for new therapists. •Ensure competency through performance competency checks. •Enhance existing or establish new safety policies and procedures. •Advocate for the profession and for systemic change.	•Participants varied greatly in years of practice experience and type of setting. •Social desirability might have affected participants’ points of view despite our efforts to minimise such impact.
Practice errors	Corrado et al., 2014	Quantitative (survey) Annali di igiene : medicina preventiva e di comunita	To explore the characteristics of the clinical risk in rehabilitation to learn more about its extent, its components, and its implications for the user.	Italy (49 private rehabilitation centres) Four different disciplines (representation between disciplines unknown) •Occupational therapy •Speech therapy •Physiotherapy •Psychomotor education	Out of a total of 556 questionnaires distributed, 493 were returned (88.6% response rate.). 21 error types were categorised into 7 macro categories: 1) errors linked to structural aspects and the rehabilitation setting; 2) errors linked to information; 3) errors linked to organisational, bureaucratic and administrative aspects; 4) errors linked to technical and professional aspects; 5) errors linked to relationship aspects; 6) errors linked to the application of and adjustment to specific current legislation and 7) miscellaneous errors. •441 respondents reported 15673 errors. On average 35 errors during their careers. Seniority of the healthcare workers analysed to be around nine and a half years, with a modal value of ten years. •Out of the 15673 errors, 75.17% occurred in outpatients’ clinics, 11.74% in other spaces, 7.06% in a gym and 5.92 in inpatient facilities and 0.09 not stated. •The consequences were mild in 40.16% of cases, while around 14% of the errors produced serious consequences. 51% produced moderate or serious consequences. •Most frequent occurring errors (38.38%) were linked to errors concerning technical and professional aspects: wrong dose errors, treatment-planning errors, and functional assessment errors. •The second highest frequency (17.41%) was linked to ‘errors linked to information’. •Errors relating to ‘organisational, bureaucratic and administrative aspects’ were 17.30% of total events. •Organisation/systems latent risk was described in greater details as: poor maintenance of equipment, lack of rehabilitation tool uniformity, inadequate identification of roles and work organisation, excessively small, unhygienic and insufficiently private therapy areas, wrong dose errors linked to local health service prescriptions, and too many services per unit of time, with the consequent impossibility to communicate with other professionals. •Other reported errors included excessive empathy and the risk of burnout.	•Professional setting with no tradition of participating in research studies, whose workers were not accustomed to reporting their errors. •Some interviewees may have doubted that their anonymity would be respected and, as a result, may have under-reported the events due to fear of their mistakes being discovered.
Pressure care	Rose and MacKenzie, 2010	Qualitative (grounded theory and semi-structured interviews) Disability and rehabilitation	The purpose of the study was to investigate the perceptions of occupational therapists about their role within pressure care and the influences on clinical decisions in this area.	Australia Occupational therapists (n=9) Diverse practice areas, including community health (n=2) and rehabilitation (n=1)	The core category was identified to be, ‘Going beyond the cushion: matching the pressure care solution to the client’. This involved the therapists (participants) to use their knowledge and experience; gather information from clients, suppliers and other health professionals; make decisions; trial equipment; follow up and evaluate their interventions and manage resources appropriately. Subcategories were identified as:•Client-centred approach •Role perceptions and expectations •Knowledge and experience •Decisions and actions •Managing resources The occupational therapy role in pressure care is shaped largely by context, role traditions, knowledge and experience. A lack of pressure care education was identified. Clearer guidance on role within pressure care is required for undergraduate educators and occupational therapy managers in the education of students and practicing therapists. Optimal outcomes for clients with pressure care needs can be achieved by improving a therapist’s skills and competence, together with cost effective methods and multi-disciplinary collaboration.	•Small study in one geographical area, the results are limited in their ability to be generalised to other occupational therapists. •Data collection was limited (one interview per participant) and the lack of additional interviews/ observations restricted data verification.

### Charting the data

Included studies were organised in Microsoft Excel and the data were extracted and charted as shown in Table 2 (Joanna, 2020). The first author completed and organised the data in the following categories:

• Risk domain;
• Author/year;
• Methodology/publication description;
• Study purpose;
• Location/sample;
• Key findings and
• Limitations (reported).

### Collating, summarising and reporting results

Content analysis of all eligible studies was conducted in two stages by the first author: a descriptive analytical approach to establish frequencies followed by thematic analysis to identify themes and patterns systematically (Braun and Clarke, 2006). This method facilitated the creation of risk domains and study categorisation therein, risk domain frequency and a summary of the risk characteristics in relation to the identified risk domains. Deciding upon the risk domain categories was achieved after a full text review of each study and team meetings to help refine the risk domain criteria. Risk characteristics were identified by the first author through thematic analysis of the results, findings and discussion sections of included studies to generate descriptive codes. These codes were stored and organised in QSR International NVivo 12. Theme generation was achieved by a collaborative process between all authors before deciding upon the risk characteristics to be reported (Braun and Clarke, 2006).

## Results

The database searches identified 2878 hits. After duplicates had been removed, 1862 were screened by title and abstract. A further 1820 were excluded which left a full text review of 42 studies, where 17 studies were excluded. No further studies were identified during a search of the reference lists of included studies. Three studies were unavailable resulting in 25 studies being included in this review. The search process is shown in Figure 1. All included studies were published between 2000 and 2019 and ten (60%) were published within the last 10 years. Of the included studies, 11 used qualitative study designs, eight used quantitative methods, three were literature reviews, two were mixed methods studies and one Delphi study.

### Assessment of methodological quality

The qualitative studies reviewed were diverse and used a variety of study designs, including grounded theory, phenomenology and secondary data analysis. The prominent data collection methods were semi-structured interview and focus group. Four main areas presented a quality concern: those were the sampling methods used, the role of researcher, decision trial auditability and trustworthiness. The sampling methods was often not described in detail and were in most cases not related to sampling redundancy; however, it was noted that achieving data saturation in relation to recruiting a sample with flexibility may not have been an objective for these studies. The role of researcher was often overlooked in respect of their level of participation and expertise. Regarding auditability concerns, decision-making trails relating to how codes of data were identified and how they were transformed into themes was not reported in detail. The four components of trustworthiness, those being credibility, transferability, dependability and confirmability were not all addressed in the majority of the studies reviewed.

The quantitative studies reviewed used three prominent study designs, those being, cross-sectional, cohort and evaluative. The quality assessment of these studies alluded to potential deficiencies in three areas, which were the sample size justification, the reliability and validity of outcome measures and the methods used in data analysis. None of the studies appraised were interventional, therefore, some of the critical appraisal tool used was not applicable. Regarding the sampling method, the sample size was not justified for the studies employing inferential statistical analysis, possible selection bias was not reported, groups were not equal in size and the sample was often not described in detail. Outcome measures were not reported in terms of their empirical validity and reliability and some studies omitted whether they used a pilot study or employed a screening process to determine whether their outcome measures or psychometric scales were reliable and valid. Additionally, the rationale for using statistical testing was rarely described and most studies reported limitations to the generalisability of their findings.

Additionally, the remaining studies, mixed method (*n*=2) and a Delphi study also presented quality concerns in the sampling method reported. One out of the three literature reviews in this study used systematic methods and these studies ranged from 2003 to 2010 which may bring concern to their current clinical relevance in relation to this study’s research objectives. The quality assessment summary of the quantitative and qualitative studies can be seen in Appendix 2.

### Risk domains

**Figure 2.**
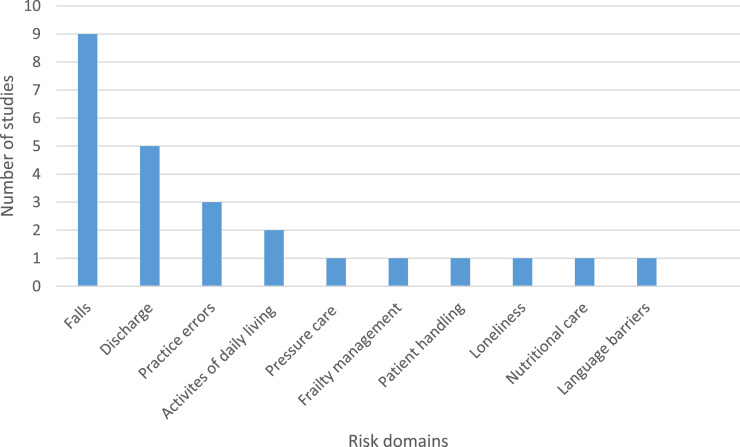
Risk domain frequencies.

With regard to the Research Aims 1 and 2, the risk domain frequencies are Falls (*n*=9), discharge (*n*=5), practice errors (*n*=3), activities of daily living (*n*=2), pressure care (*n*=1), frailty management (*n*=1), patient handling (*n*=1), loneliness (*n*=1), nutritional care (*n*=1) and language barriers (*n*=1) as shown in Figure 2. The studies that relate to falls (36%), discharge (20%) and practice errors (12%) represent the highest frequency of risk domains and contribute to 68% of the total studies included in this review.

Examination of the nature and scope (Aim 2) of the risk domains was conducted to identify the research methodologies, practice settings and the focus of the research within each risk domain, as presented on Tables 3–5. In describing the common areas of risk as risk domains, three main areas of ambiguity were identified and resolved:

• The ‘Discharge’ risk domain included those studies that focused on home visits prior to discharge. All home visits were initiated in the context of discharge; therefore, discharge became the area of risk and was categorised as the risk domain.
• The ‘Activities of daily living’ risk domain incorporated those studies which focused on assessments, interventions and the clinical reasoning of occupational therapists in determining ability during activities necessary to remain independent, safe and to live at home.
• Where multiple risk domains were identified, the aim(s) and primary focus of the study became the over-riding factor in risk domain determination.

**Table 3.** Frequency of research focus by risk domain.

Risk domain Research focus	Falls	Discharge	Practice errors	Activities of daily living	Frailty	Language barriers	Loneliness	Nutritional care	Patient handling	Pressure care
Prediction	1	1	—	—	—	—	—	—	—	—
Prevention *(including adapting strategies)*	5	—	1	—	—	—	—	—	—	—
Intervention	—	1	—	—	—	—	—	—	—	—
Clinical practice (including management of conditions)	—	1	1	—	—	—	—	—	1	—
Perceptions (including perspectives)	—	—	1	—	1	1	1	1	—	1
Risk factors	2	—	—	—	—	—	—	—	—	—
Decision-making	—	2	—	—	—	—	—	—	—	—
Assessment *(including assessment modification)*	1	1	—	1	—	—	—	—	—	—
Detection	1	—	—	—	—	—	—	—	—	—

Note: includes multiple areas of focus within a single study.

**Table 4.** Frequency of practice setting by risk domain.

Risk domain Practice setting	Falls	Discharge	Practice errors	Activities of daily living	Frailty	Language barriers	Loneliness	Nutritional care	Patient handling	Pressure care
Home (including residential homes)	2	1	—	—	1	1	—	1	—	—
Rehabilitation (including convalescence settings	4	2	3	—	—	—	—	—	—	1
Inpatient	1	1	—	—	—	—	—	—	1	—
Acute care (including acute rehabilitation*)*	—	1	—	—	—	—	—	—	—	—
Community (including community centre)	—	—	—	1	1	—	1	—	—	1
Other (including outreach or other health services	1	—	—	—	—	—	—	—	—	—

Note: includes multiple practice settings within a single study.

**Table 5.** Frequency of research methodologies by risk domain.

											
Methodology	Risk domain	Falls	Discharge	Practice errors	Activities of daily living	Frailty	Language barriers	Loneliness	Nutritional care	Patient handling	Pressure care
Qualitative	—	2	2	2	—	—	1	1	1	1	1
Quantitative	—	4	2	1	1	—	—	—	—	—	—
Mixed methods	—	1	—	—	—	1	—	—	—	—	—
Delphi	—	1	—	—	—	—	—	—	—	—	—
Literature review (including scoping review)	—	1	1	—	1	—	—	—	—	—	—

### Risk characteristics

To address Aims 2 and 3, prominent themes, and features of risk from the reviewed literature were categorised as risk characteristics. Three risk characteristics were identified: (1) Risk awareness and identifying risk, (2) decision-making under risk and (3) Improving safety.

### Risk awareness and identifying risk

Risk awareness may be defined as the acknowledgement of a condition, disability, disease, patient safety issue or a risk-prone situation that when unaddressed has the potential to cause harm. Risk identification includes best practice methods for identifying risk and/or risk factors that present safety issues or inhibit wellbeing.

Ruchinskas et al. (2001) emphasised the importance of identifying known fall risk factors to support accurate fall prediction and found cueing helped predictive accuracy and participants’ ability to identify ‘history of falls’ but not ‘advancing age’ risk factors. Ruchinskas (2003) found therapists demonstrated some predicative capability for falls, however not exceeding that of using two major predictors: ‘falls history’ and ‘presence of a neurological condition’. In contrast, Pighills et al. (2019) surveyed occupational therapists and found the majority agreed that people at a high risk of falls include those with a history of falls, visual impairment, those who are aged, have co-morbidities or had had a recent hospital visit.

Several studies have focussed on identifying and mitigating risk factors. Buri et al. (2000) found perceptual dysfunction was related to falls in older people with cognitive impairment and spatial disorientation was the most important perceptual risk factor. Xu et al. (2019) sought to adapt a falls’ prevention program for stroke survivors as they have condition-specific risk factors for falling which include hypertension medications, neurological visual disorder and post-stroke depression. Occupational therapists understand and routinely ask about pressure care needs (Mole et al., 2019) and use a client-centred approach to identify and address such issues (Rose and Mackenzie, 2010). They perceive loneliness as a psychosocial risk factor associated with higher risk of developing poor health outcomes, epitomised by social isolation, depression and physical deconditioning, lack of self-care and falls (Chana et al., 2016). Bathing for adults with physical disabilities is seen as a potential risk owing to hard, sharp surfaces and the presence of water and occupational therapists ranked the most important assessment and solution considerations as mobility, client priorities, safety factors, medical diagnosis and the availability of bathing equipment (Gooch, 2003).

Studies investigating home visits as an intervention during discharge have focused on client mobility and functional deficits, unsafe environments and risk-prone situations. Nygård et al. (2004) found that occupational therapists associate client problems during discharge home visits with inadequacies in motor, cognitive and psychological capacity and environmental hazards. Davis Aisling and Mc Clure (2019) identified home visits as potentially unsafe areas of practice for therapists, sometimes involving lone working or dangerous social situations and hazardous environments.

Best practice methods for equipment selection for safe patient handling was associated with the awareness of physical, behavioural, cognitive and perceptual characteristics of each patient, the equipment’s features, suitability and the environmental demand (Darragh et al., 2013). Barriers that inhibit patient safety and cause practice errors were investigated by Scheirton et al. (2003), Mu et al. (2011) and Corrado et al. (2014). Scheirton et al. (2003) and Mu et al. (2011) findings suggest occupational therapists consider that practice errors arise from individual and organisational failings. Corrado et al. (2014) found poor maintenance of equipment, unsuitable private therapy areas, medication errors, unrealistic time scales for services to communicate, confusion over role, inadequate organisation of workload and lack of uniformity in rehabilitation tools caused latent risk factors in organisations and their systems.

### Decision-making under risk

Risk characteristics of ‘decision-making under risk’ refer to studies that include one or more risk judgements in prevention strategies, assessments, predictions and interventions to manage risk and/or delineate the clinical reasoning in decision-making.

Clinical reasoning has been found to incorporate many perspectives including using clinical experience and learning through error. Carrier et al. (2010) asserted that decision-making components used by community occupational therapists include interactive decision-making, quick formation of solutions prior to comprehensive reasoning and dimensions of clinical reasoning used simultaneously. Additionally, integrating tacit knowledge with formal knowledge were features of this decision-making influenced by internal (personal context) and external (practice context) factors. Rose and Mackenzie (2010) found that clinical reasoning in occupational therapy pressure care was multifactorial involving client diagnosis, prognosis and collaboration. Additionally, the volume of the products, cost, equipment needs and their impact were also part of the decision-making process that often led to ‘compromise’ and ‘trial and error’ methods. In assessing for frailty, Roland et al. (2011) established that therapists would look for signs of poor judgement, impaired decision-making, limited physical function and cognitive ability to recognise and articulate needs. In the over 65 age group risk, Kinn and Galloway (2000) contend the likelihood of injury increases for those who cannot rise after a fall and found clinical experience to teach clients how to rise was the only reported method used to mitigate this risk. Scheirton et al. (2003) found learning through error was considered a valued learning experience in their study of occupational therapists’ responses to practice errors.

Deficiencies in organisational processes and approaches to therapy were found to influence decision-making under risk. Mole et al. (2019) identified that organisational failings and therapist inadequacies can affect nutritional care, specifically limited time, nutritional knowledge and financial pressure to replace carers with meal delivery support. Corrado et al. (2014) found practice errors relating to wrong dose, treatment planning and functional assessment were the most frequently reported, and organisational, bureaucratic and administrative factors were important considerations in clinical risk management.

Differing approaches to assessment were seen to influence decisions relating to risk-prone activities. Gooch (2003) found assessing bathing in adults with physical disabilities was inconsistent and not always conducive with best practice methods for determining functional ability. Telephone assessments were more frequently reported than the use of standardised assessments and over half reported using their own assessment methods and when face-to-face assessment took place it was mostly conducted without water.

Enhancing standardised methods in order to improve decision-making under risk was considered in the studies by Xu et al. (2019) and Hasegawa and Kamimura (2018), where adapting fall assessment and prevention programmes were brought more in line with the client group, culture and environmental demands. Pighills et al. (2019) found environmental home assessments and modifications for falls were affected by therapists’ confidence in and awareness of guidance, key stakeholder support, misunderstanding the value of occupational therapy, financial implications and time to complete modifications and administration. Risk prediction is an inevitable component of risk management and identifying those who may fall with a degree of predictive accuracy in the over 60 age group was found to be difficult (Ruchinskas, 2003). In contrast, Simning et al. (2019) found the predictions of the occupational and physiotherapists were veridical with discharge outcomes in older adults transitioning to home.

Ethical considerations, client-centred decision-making and client behaviour were found to be factors in mitigating risk. Following up on recommendations to review compliance to interventions and adopting a client-centred approach were found to be important components in attempting to prevent falls for those older adults living alone (Woodland and Hobson, 2003). Moats and Doble (2006) found an association between risk-taking and client-centred practice as clinical decision-making is often guided by autonomy promotion and accepting the risk a client is prepared to take. Their findings suggest autonomy promotion is subject to conflicting ethical principles, the fear of risk-taking repercussions, socio-political values, service traditions, prejudice and/or economic directives that support risk avoidance. These factors were identified to sometimes lead to inappropriate methods of care involving persuasion, coercion and intimidation (Moats, 2007). Additionally, the findings of Nygård et al. (2004) suggest a client-centred approach is tested when a client’s behaviour increases risk and Moats and Doble (2006) found client centeredness is often abandoned when clients place themselves in danger.

Systemic organisational factors were found to influence decisions and behaviours relating to risk-prone situations. Squires et al. (2019) found preventing the miscommunication of risk in the use of interpreter services engendered proactive decisions relating to the organisation of workload to ensure harm did not result from inaccurate interpretation. Chana et al. (2016) found intermediate team members considered ‘loneliness’ a relevant issue; however, managing loneliness was a low priority within the intermediate care service caused by a propensity to work only towards symptoms and functions within a traditional medical model.

### Improving safety

The ‘improving safety’ risk characteristic includes recommendations for improving risk-prone areas of practice, adaptation or modification of therapeutic tools, removal of barriers inhibiting safety, research development and organisational factors not conducive with safe practice.

Improving falls research, education, clinical supervision, and prevention programmes were seen as necessary to increase the uptake in programme participation, mitigate risk and to sustain services. Olij et al. (2017) reported the need to remove financial barriers and improve healthcare counselling and national health education. Pighills et al. (2019) called for better access to peer support and collaboration with key stakeholders. In recognition of increasing healthcare costs, Xu et al. (2019) contend that group-based falls-prevention interventions for stroke survivors such as ‘Stepping On’ could improve cost effectiveness. Hasegawa and Kamimura (2018) developed a Japanese version of the Westmead Home Safety Assessment to prevent falls in older adults and identified further research was required to improve its reliability and validity. Ruchinskas et al. (2001) and Ruchinskas (2003) contend staff education on empirically supported risk factors for falls may reduce the potential for error and improve decision-making and patient care. Kinn and Galloway (2000) found nearly half of therapists did not teach older persons how to rise from the floor after a fall. Recommendations for improvement included more teaching at undergraduate level and clinical supervision.

Factors identified to improve discharge planning and home visits included systemic organisational change, collaboration and communication between key stakeholders and client centeredness approaches. Davis Aisling and Mc Clure (2019) proposed additional time to complete visits, standardised checklists for hazard identification, further policy guidance, better transport options, occupational therapy assistant support, administrative resources and collaboration between community services and multi-disciplinary teams. Nygård et al. (2004) recommended service improvements for discharging inpatient older adults in line with their findings, which concluded the client’s wellbeing can be affected by too many workers visiting them, the adoption of follow-up visits and better communication in providing care and ordering equipment.

Alleviating inhibitive workloads and removing barriers preventing best practice, improving working relationships, assessment tools, education and research were identified in many of the studies reviewed. Roland et al. (2011) found that ameliorating the effects of a therapist’s workload could potentially improve frailty detection amongst at risk populations, facilitate prevention contingencies and response to acute cases. Squires et al. (2019) found a consensus amongst their participants that supporting clinicians to manage non-English speaking patients would potentially improve outcomes and quality of care. Chana et al. (2016) recommended improving the detection and management of loneliness within intermediate care services by addressing the following barriers: high workloads, unsatisfactory referral systems and lack of close working with social care and independent sector services. Additionally, bringing reliable brief assessments into practice, training on detecting and managing loneliness and improving working relations with key stakeholders were seen as necessary for improving services. Corrado et al. (2014) and Mu et al. (2011) recommended focusing on and advocating for systemic change which would help reduce practice errors and improve patient safety. Scheirton et al. (2003) recommended future occupational therapy research should target, explore and develop specific strategies to prevent and reduce practice errors.

Mole et al. (2019) proposed improvements in the detection and management of nutritional care including developing training aids, education on identifying nutritional risk and helping families make appropriate meal choices to prevent malnutrition. Rose and Mackenzie (2010) suggested further and clearer guidance on the occupational therapy role in pressure care for undergraduate educators and service managers to educate students and existing practitioners. Darragh et al. (2013) recommended further research relating to the development of equipment designed for therapeutic activity is crucial for therapist and client safety. Gooch (2003) found further investigation was required to determine the safety considerations for adults with physical disabilities bathing and what risk factors should be considered by occupational therapists.

## Discussion

The purpose of this scoping review was to identify the common areas of risk and their characteristics in intermediate care from an occupational therapy perspective. Twenty-five articles were reviewed comprising a range of study designs and methodological approaches. The common areas of risk have been described as risk domains and three prominent risk characteristics have been identified from the literature reviewed. In terms of methodological quality, there were some areas where quality assessment items were not reported across many or all included studies. However, all studies were found to have relevant and meaningful conclusions and, therefore, worthy of attention and significant to this study.

‘Falls’, ‘Discharge’ and ‘Practice errors’ were the most prominent risk domains accounting for seventeen (68%) of the studies reviewed, and the remaining eight studies accounted for seven risk domains (see Figure 2). There is an absence of studies that focus on the components of risk management particularly outside of the ‘Falls’ and ‘Discharge’ risk domains (see Table 3), and ‘Rehabilitation’ settings followed by ‘Home’ and ‘Community’ were the most common research locations (see Table 4). The majority of research reviewed was qualitative in nature or used descriptive quantitative survey designs (see Table 5). However, many of these studies were not about risk itself but sought to understand therapists’ perspectives of a particular area of practice that is synonymous with risk. The focus of these studies was establishing conceptual perspectives, working practices, barriers to providing care and the occupational therapy role and did not explicitly focus on how risk was mitigated in these risk domains.

The most common risk domain was ‘Falls’ accounting for nine studies and many risk characteristics in this review. This reflects falls being the major cause of disability and mortality in older people in the UK (DOH, 2001). Older adult fall prevention is complex with over 400 risk factors for falls. The risk of falling appears to increase with the number of risk factors and this requires multifactorial risk assessments across different healthcare professionals to target interventions to mitigate fall risk factors (National Institute for Health and Care Excellence, 2015). The methods of fall prevention and management in the ‘Falls’ risk domain concentrated primarily on physical, psychosocial and environmental factors and the effect on occupation was not fully explored. Woodland and Hobson (2003) found occupational therapy was underrepresented in falls literature and there was a clear gap in knowledge regarding the role that occupational therapy plays in older adults fall prevention. The role of occupational therapists working with older adults to prevent and manage falls is not exclusive to those working in specialist falls services as ‘person’, ‘environment’ and ‘occupation’ considerations align with intrinsic (personal), extrinsic (environment) and behavioural (occupation) fall risk factors (RCOT, 2020). The process of how falls risk factors are reconciled with occupational routines in intermediate care remains unclear from the literature reviewed.

The ‘Discharge’ risk domain included five studies that support discharge planning as multifactorial and subject to risk. Older adults are likely to have or develop multi-morbidity which is known to increase the likelihood of hospital admission and re-admission (Age UK, 2019) and intermediate care is essential to facilitate timely and safe discharge (NHS Benchmarking Network, 2017). Nygård et al. (2004) and Davis Aisling and Mc Clure (2019) assert home visits during discharge planning are important for identifying risk associated with problems related to a client’s physical, cognitive and psychological capacity in addition to assessing their environment for hazards. In contrast, Nygård et al. (2004) found occupational therapy interventions predominantly focus on ameliorating the effect of physical impairment by prescribing assistive equipment or environmental adaptations. Moats and Doble (2006) and Moats (2007) contend discharge planning during home visits often involves autonomy versus safety considerations in balance with professional objectives, support and resource availability and the concerns of family and carers. Whilst this review has provided insight into the styles of reasoning that factor into decision-making under risk, there is a lack of information relating to how the severity, impact and likelihood of risk is assessed to safely facilitate discharge and promote independence.

Making judgements on risk to prevent or reduce the likelihood of practice errors introduces another perspective in risk management. The focus of many studies in relation to mitigating harmful risk concentrates on therapeutic activity; however, three studies catagorised in the ‘Practice error’ risk domain explore causational factors beyond that of the individual (Scheirton et al., 2003; Mu et al., 2011; Corrado et al., 2014). Organisational risk factors can be localised or systemic and they can also impact service users disproportionately. They can relate to all aspects of an organisation including policies, procedures, the actions of staff, management of resources and the availability and provision of assistive equipment (Mu et al., 2011; RCOT, 2017). These risk factors can be latent and less obvious (Corrado et al., 2014) and their potential effect cannot be overlooked or considered beyond any responsibility to take action to mitigate their potential harm. Practice errors can cause emotional responses as they are seen against professional standards (Scheirton et al., 2003); however, their inevitability also provide opportunities to improve services. Open and honest reporting will facilitate learning through error and support of a ‘whole system’ approach to mitigate their future occurrence (Scheirton et al., 2003; RCOT, 2017).

Risk awareness and identifying risk is the first step in the risk management process. Haxby et al. (2011) assert risk awareness means that individuals and organisations can potentially prevent practice errors from causing harm to patients. Likewise, identifying risk relating to clinical, operational and financial processes is fundamental in risk management and to creating sustainable, safe and effective healthcare (Haxby et al., 2011). Making decisions under risk sometimes requires using contradictory or incomplete information making the determination of risk factors difficult. Risks are quite often viewed as socially constructed and determining the likelihood and severity of any potential event is dependent on subjective viewpoints which are influenced by many factors including heuristics and biases (Breakwell, 2007). The result of such influences can act against effective decision-making and quality of care. Many factors to reduce harmful risk, support decision-making and improve the quality of care are evident in the ‘Improving safety’ section of this review; however, education and training are prominent themes which can support decision-making under risk, improve risk management skills and help create a risk enablement culture (RCOT, 2017).

Despite the scope of risk characteristics identified, the methods of how occupational therapists assess the severity and likelihood of risk, communicate it and evaluate any outcomes from interventions relating to it, are notably absent in the literature. The National Institute for Health and Care Excellence (2017) recommends occupational therapists support positive risk-taking in intermediate care. This review did not identify any studies that explicitly focus on how occupational therapists facilitate risk enablement or positive risk-taking (RCOT, 2017). However, there are many examples of the implicit approaches occupational therapists are employing to ensure occupational dysfunction is ameliorated, harmful risk is mitigated and positive outcomes are realised.

### Implications

It was expected that there would be a paucity of research relating to risk management, including positive risk-taking, in intermediate care from an occupational therapy perspective which is why a scoping review with a broader focus was conducted. Possible reasons for this lack of information may reside in the diverse nature and approaches used in risk management and how occupational therapists use clinical and professional reasoning, informal theories and tacit knowledge to problem solve risk-prone situations. These techniques may be difficult to communicate and therefore difficult to investigate in research. However, examples of best practice methods including overcoming barriers to employing such risk management strategies that support policy and guidance have not been identified. This knowledge gap presents implications to occupational therapy student and clinical practice education. It is important to develop training programmes that are evidence based and are reflective of occupational therapy expertise in the delivery of intermediate care. This challenges future research to investigate the explicit methods of risk management and how positive risk-taking is facilitated in intermediate care by occupational therapists involved in its delivery and who are experts in their field.

### Strengths and limitations

This scoping review has been conducted using a systematic and rigorous process and has benefitted from the experience of a multi-professional research team and a comprehensive quality assessment of the studies under review. Intermediate care has different definitions and therefore, a broad and inclusive criterion was adopted. This resulted in a broad focus on different areas of practice that may not be fully representative of any specific intermediate care setting. Many studies relating to discharge were screened out as they did not meet our definition of intermediate care and these studies may have added value to this review. There were studies that included perspectives from disciplines other than occupational therapy and this must be considered in the findings of this review. Three studies were not available.

## Conclusion

This scoping review identified 10 risk domains and three areas of risk characteristics which are central to occupational therapy practice in intermediate care.

Occupational therapists predominantly seek to mitigate risk relating to a client’s symptoms, mobility and function within their environment but are aware of risk related to themselves, suboptimal systems and processes within organisations. Organisational policies and practices together with high demands for intermediate care services are not always congruent with mitigating risk relating to psychosocial phenomena such as loneliness. This can cause conflict between those providing care and service providers.

There are many examples of the implicit management of risk in relation to the positive effect of occupational therapy interventions in the ‘Decision-making under risk’ and ‘Improving safety’ risk characteristics. However, this review has found no explicit information relating to key risk management strategies including how the likelihood and severity of risk is assessed and how positive risk-taking is facilitated. Likewise, there is a lack of occupational focus and therefore a gap in knowledge as to how risk is embraced and reconciled with the value and need for occupation for those accessing intermediate care services. Successful positive risk-taking is dependent on effective risk management skills. Future research must focus on all aspects of risk management and how positive risk-taking is factored into occupational therapy interventions in relation to older adult intermediate care in support of the current policy and guidance.

## Key findings

• 10 risk domains were identified, ‘Falls’ being the most common.
• Three prominent risk characteristics were reported.
• Managing occupation in relation to risk-taking strategies was implicit within the literature reviewed.

## What this study has added

This study has mapped the current literature relating risk in intermediate care from an occupational therapy perspective, providing insight into risk within the service to further knowledge and research direction.

## Appendix 2. A quality assessment summary of the quantitative and qualitative studies.

**Table:**

Quantitative								
Studies Quality assessment questions	Gooch, 2003	Davis Aisling and McClure, 2019	Simning et al., 2019	Kinn and Galloway, 2000	Ruchinskas et al., 2001	Ruchinskas, 2003	Hasegawa and Kamimura, 2018	Corrado et al., 2014
1. Was the study purpose clearly stated?	✓	✓	✓	✓	✓	✓	✓	✓
2. Was relevant background literature reviewed?	✓	✓	✓	✓	✓	✓	✓	✓
3. Was the design appropriate for the study question?	✓	✓	✓	✓	!	✓	✓	✓
4. Were any bias(es) identified or considered?	✓	✓	✓	✓	✓	!	!	!
5. Was the sample/sampling process described in detail?	!	✓	✓	×	!	!	!	!
6. Was sample size justified?	×	N/A	×	N/A	N/A	×	×	N/A
7. Were the outcome measures reliable?	!	!	!	!	!	✓	✓	!
8. Were the outcome measures valid?	!	!	!	!	!	✓	✓	!
9. Intervention was described in detail?	N/A	N/A	N/A	N/A	N/A	N/A	N/A	N/A
10. Contamination was avoided?	N/A	N/A	N/A	N/A	N/A	N/A	N/A	N/A
11. Cointervention was avoided?	N/A	N/A	N/A	N/A	N/A	N/A	N/A	N/A
12. Results were reported in terms of statistical significance?	✓	N/A	✓	N/A	N/A	✓	✓	N/A
13. Were the analysis method(s) appropriate?	!	!	✓	✓	!	✓	!	!
14. Clinical importance was reported?	✓	✓	!	✓	✓	✓	✓	✓
15. Dropouts were reported?	N/A	N/A	✓	N/A	N/A	✓	N/A	N/A
16. Conclusions were appropriate given study methods and results?	✓	✓	✓	✓	✓	✓	✓	✓

Qualitative											
Studies Quality assessment questions	Moats, 2007	Nygård et al., 2004	Buri et al., 2000	Xu et al., 2019	Squires et al., 2019	Chana et al., 2016	Mole et al., 2019	Darragh et al., 2013	Scheirton et al., 2003	Mu et al., 2011	Rose and MacKenzie, 2010
1. Was the study purpose clearly stated?	✓	✓	✓	✓	✓	✓	✓	✓	✓	✓	✓
2. Was relevant background literature reviewed?	✓	✓	✓	✓	✓	✓	✓	✓	✓	✓	✓
3. Was the design appropriate for the study question?	✓	✓	✓	✓	✓	✓	✓	✓	✓	✓	!
4. Was the sample/sampling process described in detail?	✓	**!**	✓	**!**	✓	!	!	!	✓	!	!
5. Was the sampling method appropriate to the study purpose or research question?	**!**	**!**	✓	**!**	**!**	✓	✓	!	✓	!	✓
6. Was sampling done until redundancy in data was reached?	×	×	×		**N/A**	×	×	×	×	×	×
7. Clear and complete description of the site and participants?	✓	✓	✓	**!**	✓	✓	✓	✓	✓	✓	✓
8. Clear role of the researcher and their relationship to the participants?	×	✓	✓	**!**	**!**	×	✓	**!**	**!**	×	**!**
9. Was there sufficient information to understand the data collection?	✓	✓	✓	✓	✓	✓	✓	✓	✓	✓	✓
10. Procedural rigour was used in data collection strategies?	✓	✓	!	✓	✓	✓	✓	✓	✓	✓	✓
11. Data analyses were inductive and appropriate?	✓	✓	✓	✓	✓	✓	!	✓	✓	✓	✓
12. Findings were consistent with and reflective of data?	✓	✓	✓	✓	✓	✓	✓	✓	✓	✓	✓
13. Decision trail developed?	×	×	×	×	×	×	×	×	×	×	×
14. For auditing, is the process of analysing the data was described adequately?	✓	✓	!	✓	✓	!	✓	!	!		✓
15. Did a meaningful picture of the phenomenon under study emerge?	✓	✓	✓	✓	✓	✓	✓	✓	✓	✓	✓
16. Was there evidence of the four components of trustworthiness?	×	×	×	×	×	×	×	×	✓	✓	×
17. Conclusions were appropriate given the study findings?	✓	✓	✓	✓	✓	✓	✓	✓	✓	✓	✓
18. The findings contributed to theory development and future OT practice/research?	✓	✓	✓	✓	✓	✓	✓	✓	✓	✓	✓

*Key*: ✓ = yes; × = no; ! = unclear or not reported and N/A = not applicable.
